# Internal Nitrogen Pools Shape the Infection of *Aureococcus anophagefferens* CCMP 1984 by a Giant Virus

**DOI:** 10.3389/fmicb.2020.00492

**Published:** 2020-03-25

**Authors:** Eric R. Gann, Brennan J. Hughes, Todd B. Reynolds, Steven W. Wilhelm

**Affiliations:** Department of Microbiology, College of Arts and Sciences, The University of Tennessee, Knoxville, Knoxville, TN, United States

**Keywords:** Mimiviridae, nutrients, brown tide, virus ecology, infection

## Abstract

The pelagophyte *Aureococcus anophagefferens* blooms annually in shallow bays around the world, where it is hypothesized to outcompete other phytoplankton in part by using alternative nitrogen sources. The high proportion of natural populations that are infected during the late stages of the bloom suggest viruses cause bloom collapse. We hypothesized that the Aureococcus anophagefferens Virus (AaV) infection cycle would be negatively influenced in cultures acclimated to decreasing external nitrogen conditions, but that the real-time external nitrogen concentration would not influence the infection cycle. Cultures acclimated in ${\text{NO}}_{3}^{-}$ concentrations (0.0147 mM; N:P = 0.1225) that showed reduced end point cell abundances, forward scatter (a proxy for size) and red fluorescence (a proxy for chlorophyll *a*), also produced fewer viruses per cell at a slower rate. Decreasing the external concentration of nitrogen post infection did not alter burst size or time to lysis. These data suggest that the nitrogen used for new viral progeny is present within host cells at the time of infection. Flow cytometric data of an infection cycle showed a reduction in red fluorescence around twelve hours post infection, consistent with degradation of nitrogen-rich chloroplasts during the infection cycle. Using cell and virus quota estimates, we determined that *A. anophagefferens* cells had sufficient nitrogen and carbon for the lower ranges of burst sizes determined but did not contain enough phosphorous. Consistent with this observation, expression of nitrate and sugar transporters did not increase in the publicly available transcriptome data of the infection cycle, while several phosphorus transporters were. Our data demonstrate that dynamics of viruses infecting *Aureococcus* over the course of a bloom is dictated by the host cell state upon infection, which is set *a priori* by external nutrient supplies.

## Introduction

Since the accidental discovery of the ‘giant’ Mimivirus, viruses with particle sizes and genome sizes larger than many prokaryotic organisms have been isolated and are believed to belong to a monophyletic group termed the Nucleocytoplasmic large dsDNA viruses (NCLDVs) (Wilhelm et al., 2017). The increased genetic potential within these genomes allows for less dependence on the host during the infection cycle. This is evident in the production of new viruses by cytoplasmic virus factories created after infection that do not rely on the host nucleus to begin transcription (Fabre et al., 2017), as well as by the presence of viral proteins to modulate translation (Abrahao et al., 2018; Mizuno et al., 2019). This shifting paradigm, that viruses are more independent than originally believed, has led to the idea that the infected cell, sometimes termed “the virocell,” is an independent entity different from an uninfected host (Forterre, 2011). This is perhaps best illustrated by the novel products produced through a combination of host and virally encoded genes (Rosenwasser et al., 2014).

The environment the virocell is in can drastically alter the phenotype and growth parameters of the virus. Culturing amoeba hosts without bacteria for 150 generations caused the production of Mimivirus without fibrils with a reduced genome (Boyer et al., 2011). Quenching oxidative stress produced during infection of *Emiliania huxleyi* prevents new viruses from being produced (Sheyn et al., 2016). To this end, the environment the cell is in before becoming infected is important as it shapes the physiological state of the host. Most viruses accumulate the building blocks for new progeny by some combination of degrading current host stores (i.e., the nuclear genome for nucleotides) (Agarkova et al., 2006) and driving continued uptake of nutrients from the external environment (Waldbauer et al., 2019).

Changing extracellular conditions thus could play a crucial role(s) in environments where viruses are hypothesized to be important in the control of host populations. An important example of this are the blooms of eukaryotic algae in which viruses can be the sole cause of bloom collapse (Brussaard et al., 2005; Vardi et al., 2012) and have been implicated in bloom prevention (Haaber and Middelboe, 2009). The pelagophyte *Aureococcus anophagefferens* blooms annually in shallow, well-mixed bays in several locations around the world, resulting in severe light attenuation from high cell densities (>10^6^ cells mL^–1^) and apparent toxicity to bivalves that amount to millions of dollars in losses (Gobler and Sunda, 2012). A major driver of the bloom demise has been hypothesized to be large (∼190 nm), icosahedral viruses, as a high proportion of cells are visibly infected at the time of the collapse (Gastrich et al., 2004). One of these viruses, Aureococcus anophagefferens Virus (AaV), has been isolated and partially characterized, revealing it belongs to the Mimiviridae family of viruses which house some of the largest viruses known to science (Wilhelm et al., 2016). Although infected cells within natural populations have been seen since the initial description of the species (Sieburth et al., 1988), how the changing environment over the course of the bloom influences the infection cycle is understudied. Variable abiotic or biotic conditions (i.e., low temperature, low light) that may reduce the efficiency of the AaV infection (Gobler et al., 2007), could influence the succession of bacteria or other eukaryotic algae via altered release of dissolved organic matter (Gobler et al., 1997), as well as the nutrients contained within free virus particles (Jover et al., 2014).

To understand ecosystem constraints on the virus-mediated lysis of *A. anophagefferens* blooms, we examined the effect of decreasing nitrogen concentrations on the infection cycle of AaV. *A. anophagefferens* blooms form when inorganic nitrogen concentrations are drawn down and organic nitrogen concentrations are elevated (Gobler and Sunda, 2012). *A. anophagefferens* can utilize many different nitrogen compounds (Berg et al., 2002, 2008), and the assimilation of alternative organic nitrogen sources allows this alga to avoid nitrogen-limitation when other organisms become growth-limited (Gobler et al., 2004; Wurch et al., 2019). The continued use of this alternative nitrogen pool draws this pool down (Lomas et al., 1996; Gobler et al., 2011), altering the N:P ratio in the environment. In lab experiments with other algal virus systems, infection during low nitrogen conditions reduced burst sizes and increased the length of the infection cycle (Cheng et al., 2015; Maat and Brussaard, 2016), though viral production is not always stimulated by nitrogen additions to natural populations (Bratbak et al., 1993; Motegi and Nagata, 2007). In this study, we wanted to determine the influence of acclimation of decreasing nitrate concentrations (therefore decreasing N:P ratios) as cells infected at different points of natural bloom will have been exposed to differing concentrations. In parallel we wanted to determine whether the virocell would be influenced by the external nitrogen concentration, as this is dependent on the system (Fowler and Cohen, 1948; Wikner et al., 1993). We demonstrated that the AaV infection cycle was negatively influenced by host acclimation to ${\text{NO}}_{3}^{-}$ concentrations that reduced growth and cellular fluorescence, but that altering the N:P ratio after the start of infection had no effect. Our observations suggest that cellular pools of nitrogen were more important than external ones after the initiation of infection.

## Materials and Methods

### Culture Conditions

Non-axenic *A. anophagefferens* CCMP1984 cultures were grown at 19°C, on a 14:10 light dark cycle at 100 μmol photons m^–2^ s^–1^ in modified ASP_12_A growth medium (Gann, 2016). Cultures were acclimated to four different ${\text{NO}}_{3}^{-}$ concentrations (1.47, 0.735, 0.147, 0.0147 mM) (Table 1) by at least three successive transfers at each ${\text{NO}}_{3}^{-}$ concentration prior to experimentation. Preliminary efforts demonstrated that *A. anophagefferens* would not grow at concentrations less than 0.0147 mM so it was chosen as our lowest condition. Previous studies have demonstrated that culturing *A. anophagefferens* in higher concentrations of a sole nitrogen source (0.030 mM NH_4_^+^) cause physiologically and transcriptional differences compared to replete conditions (Frischkorn et al., 2014). To prevent nutrient carry over, cultures were pelleted (2,000 × *g*, 5 min), and resuspended in fresh ASP_12_A with the respective ${\text{NO}}_{3}^{-}$ concentration. The AaV lysate used to infect cultures was concentrated 50-fold from freshly lysed cultures using a Lab-scale Tangential Flow Filtration System (Fisher Scientific, Waltham, MA, United States) equipped with a Durapore^TM^ 30 kDa Pellicon XL Filter (MilliporeSigma, Burlington, MA, United States). To deplete lysates of nitrate, the concentrate was diluted 20-fold with ASP_12_A without added nitrate, and then viruses re-concentrated, before being passed through a 0.45-μm pore-size syringe filter (Millex-HV PVDF, Millipore Sigma, Burlington, NJ, United States). Less than 0.5 mL of the re-concentrated lysate was added to cultures (20 mL) for each experiment.

**TABLE 1:** Concentrations of ${\text{NO}}_{3}^{-}$ and ${\text{PO}}_{4}^{3-}$ of the different growth media.

Percent ${\text{NO}}_{3}^{-}$ of ASP_12_A	[${\text{NO}}_{3}^{-}$]	[${\text{PO}}_{4}^{3-}$]	N:P
100%	1.47 mM	0.12 mM	12.25
50%	0.735 mM	0.12 mM	6.125
10%	0.147 mM	0.12 mM	1.225
1%	0.0147 mM	0.12 mM	0.1225

### Enumeration of *A. anophagefferens* and AaV

Before enumerating concentrations, 1 mL of cultures were either directly fixed in 0.5% glutaraldehyde (for *A. anophagefferens* enumeration) or first passed through a 0.45-μm pore-size syringe filter (Millex-HV 0.45 μm nominal pore-size PVDF, Millipore Sigma, Burlington, NJ, United States) before fixation (for AaV enumeration). Although filtering cultures for enumeration of viruses reduced the concentration of viruses (∼43%) (Supplementary Figure 1), we wanted to reduce clumping or adsorption of viruses to cellular material. Fixed samples were stored at 4°C for at least 30 min before being measured on a FACSClibur flow cytometer (Becton, Dickinson and Company, Franklin Lakes, NJ, United States). *A. anophagefferens* populations were gated on red chlorophyll *a* fluorescence and forward scatter (Moniruzzaman et al., 2018). Mean forward scatter (FSC-H), side scatter (SSC-H), and red fluorescence (FL3-H) for each gated *A. anophagefferens* populations was also recorded. These act as proxies for cell size, cellular granularity, and chlorophyll *a* autofluorescence, respectively.

AaV was enumerated as described previously (Brussaard, 2004; Brown and Bidle, 2014). Briefly, the fixed AaV samples were diluted 100-fold in ASP_12_A and then inoculated with a 10,000-fold diluted SYBR Green DNA stain (Lonza, Basel, Switzerland), before being incubated at 80°C for 10 min. AaV populations were gated on SYBR green emission (excitation: 520 nm) and side scatter (Supplementary Figure 2). To determine host and virus concentrations a known concentration of diluted FluoSphere Carboxylate-Modified Microspheres, 1.0 μm, yellow-green fluorescent (505/515) (Invitrogen, Waltham, MA, United States) was added to each sample before being ran for one minute.

### Experimental Design

To determine the influence of nitrate concentration acclimation, *A. anophagefferens* cultures in late logarithmic growth and pre-acclimated to the four different nitrate concentrations (Table 1) were collected by centrifugation (2,000 × *g*, 5 min). Cell pellets were resuspended in media containing the respective ${\text{NO}}_{3}^{-}$ concentration and replicates pooled to concentrate cell densities ∼50-fold. To observe the entire growth curve, four 20 mL biological replicates were inoculated to a final cell density between 5 × 10^4^ and 1 × 10^5^ mL^–1^. To assess differences in the physiological state of the cells due to the different ${\text{NO}}_{3}^{-}$ concentrations, the mean of various flow cytometry parameters (FSC-H, SSC-H, and FL3-H) of the gated *A. anophagefferens* cells was averaged on 2 days during the middle of logarithmic growth (days 6 and 7). Doubling time of the cultures was calculated using the following equation, with days three and eight as time_N_ and time_N_0__, respectively:

$$\mathrm{Doubling}\,\mathrm{time}\;=\frac{t\,i\,m\,e_{N}-t\,i\,m\,e_{N_{o}}}{\left(\log\left(c\,o\,n\,c\,e\,n\,t\,r\,a\,t\,i\,o\,n_{N}\right)-\log\left(c\,o\,n\,c\,e\,n\,t\,r\,a\,t\,i\,o\,n_{N_{0}}\right)\right)/\log\left(2\right)}$$

To determine the influence of acclimation to different nitrate concentration on the AaV infection cycle, multiple cultures at late logarithmic growth that were acclimated to the four different nitrate concentrations (Table 1) were first pelleted by centrifugation (2,000 × *g*, 5 min). Cell pellets were resuspended in media containing the respective ${\text{NO}}_{3}^{-}$ concentration and pooled to concentrate the cultures ∼50-fold. Five biological replicates (20 mL) were inoculated to a final cell density at 10^6^ cells mL^–1^ and a multiplicity of infection (m.o.i) ∼100 particles per cell. Uninfected controls were also done in five biological replicates. A high multiplicity of infection was used as only a small fraction of AaV are infectious and infecting cultures with m.o.i’s between ∼20 and 100 allows for lysis of the majority of cells (>99%) within 3 days (Gann et al., 2020). Cultures were infected at the start of the light cycle. We collected samples immediately upon infection, and then every 4 h for 24 h, and 48 and 72 h post infection. Sampling throughout the first 24 h allowed for the adsorption rate of AaV to be determined as described previously (Cottrell and Suttle, 1995) using the following formula:

$$A\,d\,s\,o\,r\,p\,t\,i\,o\,n\,r\,a\,t\,e=\frac{a}{N}$$

where *a* is the slope of the linear regression of the natural log of free viruses over the course of the first 24 h and *N* is equal to the concentration of *A. anophagefferens.* The number of viruses produced per cell lyses, or burst size, was calculated using the following formula:

$$B\,u\,r\,s\,t\,s\,i\,z\,e=\frac{v\,i\,r\,u\,s\,c\,o\,n\,c\,e\,n\,t\,r\,a\,t\,i\,o\,n_{N}-v\,i\,r\,u\,s\,c\,o\,n\,c\,e\,n\,t\,r\,a\,t\,i\,o\,n_{N_{0}}}{c\,e\,l\,l\,c\,o\,n\,c\,e\,n\,t\,r\,a\,t\,i\,o\,n_{N_{0}}-c\,e\,l\,l\,c\,o\,n\,c\,e\,n\,t\,r\,a\,t\,i\,o\,n_{N}}$$

The host and virus concentrations at time *N*_0_ is when free viruses did not decline further: 24 h for 1.47, 0.735, and 0.147 mM ${\text{NO}}_{3}^{-}$ cultures, and 48 h for the 0.0147 mM ${\text{NO}}_{3}^{-}$ cultures. Time *N* was the end of the experiment, 72 h. Changes in basic cellular physiology during the infection cycle were determined through various flow cytometry parameters of the gated *A. anophagefferens* cells including forward scatter (FSC-H), side scatter (SSC-H), and red fluorescence (FL3-H).

To determine the influence of external nitrate concentration on the AaV infection cycle, multiple 1.47 mM ${\text{NO}}_{3}^{-}$ acclimated cultures at late logarithmic growth were first pelleted by centrifugation (2,000 × *g*, 5 min). Cell pellets were resuspended in ASP_12_A with no added nitrate and pooled to concentrate the cultures ∼50-fold. Immediately before infection, ${\text{NO}}_{3}^{-}$ was added to the cultures (final cell density = 1 × 10^6^ mL^–1^) at three different concentrations (1.47 mM, 0.0147 mM, and no added ${\text{NO}}_{3}^{-}$). Five biological replicates were infected at a m.o.i ∼20 particles per cell in 20 mL, where five uninfected controls were brought up to 20 mL with ASP_12_A with no nitrate. Samples were collected each day for 4 days. Burst sizes were calculated as described above, using time points of 1 and 4 days for times *N* and *N*_*o*_, respectively.

### Statistical Analyses

Statistical analyses were performed using Prism 7.03 (GraphPad, San Diego, CA, United States). To compare differences between treatments a one-way ANOVA was used followed by Tukey’s HSD *post hoc* testing.

## Results

### The Effects of Nitrate Concentration Acclimation on *A. anophagefferens* Growth

**FIGURE 1:**
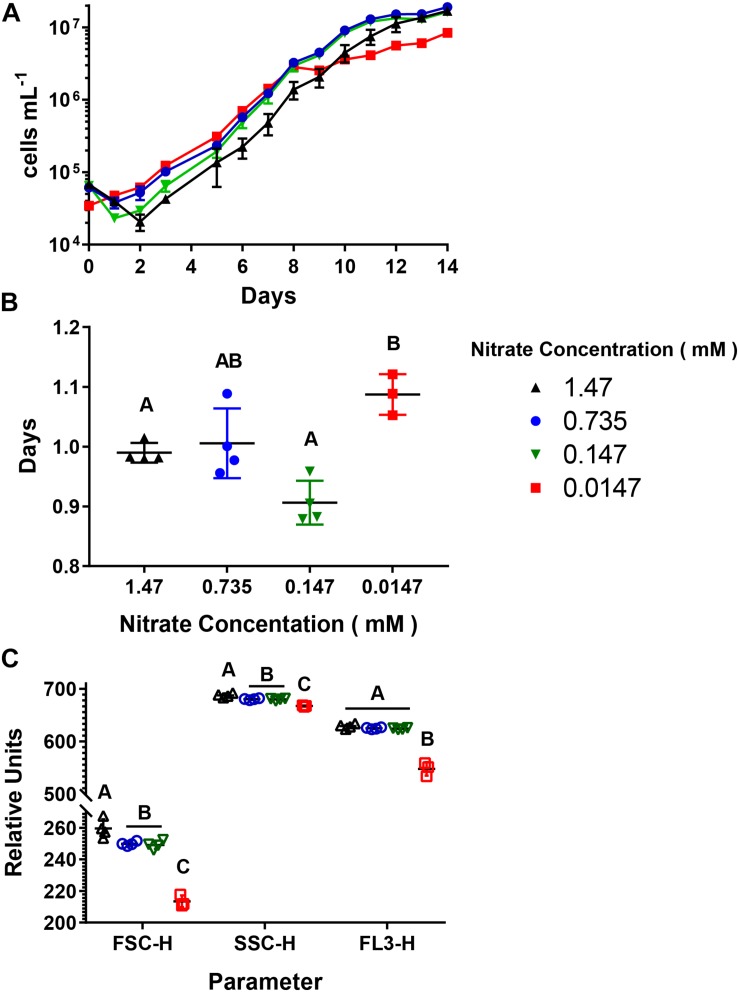
Growth characteristics of *Aureococcus anophagefferens* acclimated to decreasing ${\text{NO}}_{3}^{-}$ concentrations. **(A)** *A. anophagefferens* concentration over time. **(B)** Doubling times of *A. anophagefferens*. **(C)** Flow cytometry averages of two consecutive days of *A. anophagefferens* populations during logarithmic growth. FSC-H = forward scatter, SSC-H = side scatter, and FL3-H = chlorophyll *a* fluorescence. The different nitrate conditions are represented by the following colors and shapes: 1.47 mM – black upward triangles, 0.735 mM – blue circles, 0.147 mM – green downward triangles, and 0.0147 mM – red squares. Letters indicate that the two values being compared are not significant (*p* > 0.05). Tables with significance values are Supplementary Tables 1 and 2 for data for Figures B and C, respectively. Points are for *n* = 4 biological replicates ±SD.

Cultures were acclimated to four different ${\text{NO}}_{3}^{-}$ concentrations (1.47, 0.735, 0.147, and 0.0147 mM), with a constant ${\text{PO}}_{4}^{3-}$ concentration (0.12 mM), producing environments with decreasing N:P ratios (Table 1). There was a difference in the length of the logarithmic phase of growth as well as the final abundance of cells reached between the 0.0147 mM acclimated cultures and the other treatments. For the 1.47, 0.735, and 0.147 mM ${\text{NO}}_{3}^{-}$ acclimated cultures, the length of the logarithmic phase of growth was 9–10 days (Figure 1A), and there was no significant difference in end point abundances which were: 1.7 × 10^7^ cells mL^–1^ (SD = 9.7 × 10^5^), 1.9 × 10^7^ cells mL^–1^ (SD = 8.7 × 10^5^), 1.6 × 10^7^ cells mL^–1^ (SD = 1.4 × 10^5^), respectively (Supplementary Table 1). The 0.0147 mM ${\text{NO}}_{3}^{-}$ acclimated culture had a final cell abundance of half (8.5 × 10^6^ cells mL^–1^, SD = 9.7 × 10^5^) the three higher acclimation concentration cultures at the end of the 14-day experiment, with the logarithmic phase being 1–2 days shorter (Figure 1A). The four different acclimating ${\text{NO}}_{3}^{-}$ concentrations did not greatly influence the doubling time of the cultures, with all cultures having doubling times between 0.99 and 1.09 days (Figure 1B and Supplementary Table 2). To further understand how decreasing N:P ratios influences *A. anophagefferens* cells, we used parameters measured during flow cytometry (Figure 1B). The relative values of forward scatter (FSC-H), side scatter (SSC-H), and chlorophyll *a* autofluorescence (FL3-H) of all cells gated as *A. anophagefferens* were averaged during two consecutive days in mid-logarithmic growth (Figure 1C). The 0.735 and 0.147 mM ${\text{NO}}_{3}^{-}$ acclimated cultures had FSC-H, SSC-H, and FL3-H values that were not significantly different from one another (Supplementary Table 3). Cultures acclimated to 1.47 mM ${\text{NO}}_{3}^{-}$ had a slight, but significant increase for FSC-H and SSC-H, whereas there was statistically no difference with FL3-H compared to the 0.735 and 0.147 mM ${\text{NO}}_{3}^{-}$ acclimated cultures (Supplementary Table 3). There was a significant decrease in all three parameters for the 0.0147 mM ${\text{NO}}_{3}^{-}$ acclimated cultures compared to the other three (Supplementary Table 3).

### The Effects of Nitrate Concentration Acclimation on the AaV Infection Cycle

**FIGURE 2:**
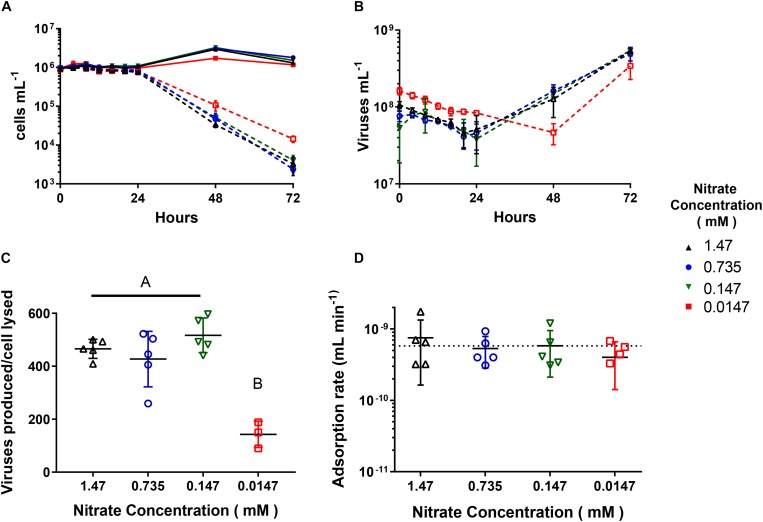
AaV infection cycle dynamics of *Aureococcus anophagefferens* acclimated to decreasing ${\text{NO}}_{3}^{-}$ concentrations. **(A)** *A. anophagefferens* and **(B)** AaV concentrations over the 3-day experiment. **(C)** AaV produced per host cell lysed. **(D)** Adsorption rates of AaV as calculated as previously reported (Cottrell and Suttle, 1995). The dotted line represents the adsorption rate (−5.8 × 10^–10^ mL min^–1^) previously reported for AaV (Brown and Bidle, 2014). The different nitrate conditions are represented by the following colors and symbols: 1.47 mM – black upward triangles, 0.735 mM – blue circles, 0.147 mM – green downward triangles, and 0.0147 mM – red squares. Filled in symbols with solid connecting lines represent uninfected controls while open symbols with dashed lines represent infected cultures. Letters indicate that the two values being compared are not significant (*p* > 0.05). Tables with significance values are Supplementary Tables 4 and 5 for data for Figures C and D, respectively. Points are for *n* = 5 biological replicates ±SD.

When we infected cultures acclimated to the four ${\text{NO}}_{3}^{-}$ concentrations with AaV at an m.o.i of ∼100, only the 0.0147 mM ${\text{NO}}_{3}^{-}$ acclimated cultures showed differences in infection dynamics. The 1.47, 0.735, and 0.147 mM ${\text{NO}}_{3}^{-}$ acclimated cultures all had similar trends as previously reported (Rowe et al., 2008; Brown and Bidle, 2014), where new viruses were produced and cells lysed after 24 h (Figures 2A,B), while cell lysis and virus production was delayed 1 day for the 0.0147 mM acclimated culture. The burst sizes for the 1.47 mM (465.8 ± 32.2 viruses produced cell lysed^–1^), 0.735 mM (427.1 ± 93.7 viruses produced cell lysed^–1^), and 0.147 mM (465.8 ± 32.2 viruses produced cell lysed^–1^) ${\text{NO}}_{3}^{-}$ acclimated cultures were not significantly different from one another. These treatments however, had a burst size ∼3× greater than the 0.0147 mM ${\text{NO}}_{3}^{-}$ acclimated cultures (142.6 ± 40.4 viruses produced cell lysed^–1^, Figure 2C and Supplementary Table 4). There were no differences in the adsorption rates due to ${\text{NO}}_{3}^{-}$ concentration (Figure 2D and Supplementary Table 5). We used parameters measured during flow cytometry to look at changes between infected and uninfected cells over the first day 24 h of the infection cycle (Supplementary Figure 3). All uninfected populations increased in FSC-H, and FL3-H, while all but the 0.0147 mM ${\text{NO}}_{3}^{-}$ acclimated culture increased in SSC-H. The FSC-H and SSC-H of infected cells either remained the same or decreased slightly (<5%) (Supplementary Figures 3A,B). The FL3-H values remained the same for the first twelve hours of the infection cycle but then decreased between 6.8 and 9.5% for all the ${\text{NO}}_{3}^{-}$ concentrations (Supplementary Figure 3C).

### The Effect of External Nitrogen on Concentration on the AaV Infection Cycle

**FIGURE 3:**
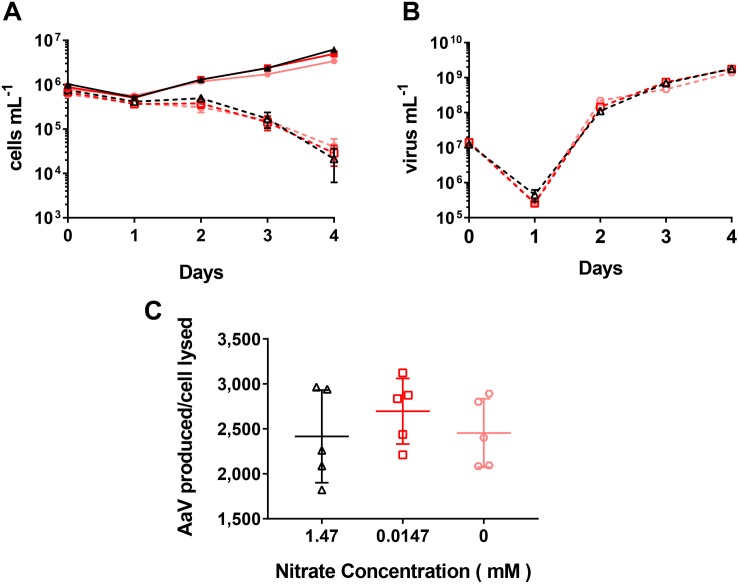
AaV infection cycle dynamics of *Aureococcus anophagefferens* acclimated to 1.47 mM ${\text{NO}}_{3}^{-}$, with different concentrations of ${\text{NO}}_{3}^{-}$ added back post infection. **(A)** *A. anophagefferens* and **(B)** AaV concentrations over the 4-day experiment. **(C)** AaV produced per host cell lysed. The different nitrate conditions added back are represented by the following colors and symbols: 1.47 mM – black upward triangles, 0.0147 – red squares, and 0.0 mM = light red hexagons. Filled in symbols with solid connecting lines represent uninfected controls while open symbols with dashed lines represent infected cultures. Letters indicate that the two values being compared are not significant (*p* > 0.05). The table with significance values is Supplementary Table 7 for data for Figure C. Points are for *n* = 5 biological replicates ±SD.

To determine whether the external nitrate concentration altered viral production, we collected *A. anophagefferens* cells grown at 1.47 mM, infected cultures at an m.o.i ∼20, then added back variable ${\text{NO}}_{3}^{-}$ concentrations (1.47, 0.0147 or 0 mM) (Figure 3). In controls, the end point abundance after the 4 days significantly decreased with reduced ${\text{NO}}_{3}^{-}$ concentrations (Supplementary Table 6). The final cell abundances were 6.2 × 10^6^ cells mL^–1^ (SD = 3.9 × 10^5^), 5.0 × 10^6^ cells mL^–1^ (SD = 5.9 × 10^5^), and 3.4 × 10^6^ cells mL^–1^ (SD = 3.1 × 10^5^), for cultures that had 1.47, 0.0147, and 0 mM ${\text{NO}}_{3}^{-}$ added back, respectively. There was also a decrease in FL3-H for the control 0 and 0.0147 mM cultures compared to the 1.47 mM cultures (Supplementary Figure 4). Although there was a hindrance to the growth of control cultures, infecting cultures with decreasing ${\text{NO}}_{3}^{-}$concentrations added back did not change the length of time for cells to lyse (Figure 3A) or new viruses to be produced (Figure 3B) or decrease the burst size (Figure 3C and Supplementary Table 7).

## Discussion

### The Effects of Nitrate Acclimation on *A. anophagefferens* Growth and the AaV Infection Cycle

The effect of acclimation of *A. anophagefferens* cultures to decreasing ${\text{NO}}_{3}^{-}$ concentrations, was only seen in the lowest concentration (0.0147 mM), where there was a decreased biomass accumulation and end point cell abundance. These acclimated cultures appeared to be physiologically different from the other cultures as there was a reduction in forward scatter, side scatter, and red fluorescence, as detected by flow cytometry. Our observations suggest that the cells in the lowest ${\text{NO}}_{3}^{-}$ concentrations are smaller (based on forward scatter) and have less chlorophyll *a* per cell (based on red fluorescence). In previous studies of various *A. anophagefferens* strains the maximum efficiency of photosystem II (*F*_v_/*F*_m_) is reduced in lower or limiting nitrogen concentrations (Berg et al., 2008; Dong et al., 2014; Frischkorn et al., 2014), and *in vivo* fluorescence per cell is also reduced (Dong et al., 2014). Experimental data is also supported by transcriptomes of *A. anophagefferens* CCMP1850 in low nitrogen environments, where there is a down regulation of light harvesting complexes and an increase in proteases and peptidases (Frischkorn et al., 2014). The reduction of red fluorescence or chlorophyll *a* per cell has been seen in other species of algae as well, leading to a reduced *F*_v_/*F*_m_ (Berges and Falkowski, 1998; Maat et al., 2016a; Maat and Brussaard, 2016), and was hypothesized that this reduction in chlorophyll *a* content decreases the nitrogen requirement within the cell (Maat and Brussaard, 2016).

Similar, to the growth kinetics of cultures, only infections of the 0.0147 mM ${\text{NO}}_{3}^{-}$ acclimated cultures were influenced negatively by the nitrate concentration, with a reduction in burst size and a prolonged production of new viruses. A prolonged infection cycle has been seen in multiple systems due to growth in lower nitrogen concentrations (Bratbak et al., 1998; Maat and Brussaard, 2016), as has the reduced burst size (Bratbak et al., 1998; Cheng et al., 2015; Maat and Brussaard, 2016). We sampled multiple times throughout the first 24 h of the experiment to detect differences in infected populations by flow cytometry and determined the adsorption rates. There was no difference in adsorption rates between any of the acclimated ${\text{NO}}_{3}^{-}$ concentration treatments. The average adsorption rate determined in this study (5.9 × 10^–10^ mL min^–1^) agrees with that previously reported in this system (5.8 × 10^–10^ mL min^–1^) (Brown and Bidle, 2014). The *Chlorella variabilis* NC64A infecting Paramecium bursaria Chlorella Virus, PBCV-1, adsorption rate was also not influenced by altered less than optimum C:P ratios (Clasen and Elser, 2007), suggesting that although algal cells differ physiologically under non-optimal nutrient conditions, viruses are able to adsorb with the same efficiency.

Forward scatter and side scatter remain constant over the course of the infection cycle, while red fluorescence begins to decrease around 12 h post-infection. A reduction in red fluorescence has been detected in other virus-host systems (Lawrence et al., 2006; Lonborg et al., 2013) at specific points in the infection cycle, depending on the virus (Lawrence et al., 2006). This correlates with a reduction in chlorophyll *a* and *F*_v_/*F*_m_ over the course of the infection cycle (Schatz et al., 2014). Measurements for *F*_v_/*F*_m_ have not been collected during the *Aureococcus* infection cycle, but daily measurements for control and infected cultures have shown an initial increase compared to control cultures then a drastic decrease (Gobler et al., 2007). In both natural populations and lab cultures, when many viruses are present in the cells there is either no chloroplast or it appears to be much smaller than uninfected cells (Sieburth et al., 1988; Gastrich et al., 2004; Rowe et al., 2008). More work is needed to determine whether this reduction in chloroplast size corresponds to the decrease in red fluorescence beginning around twelve hours post infection.

It was hypothesized that the differences in burst sizes in cells grown in low nitrogen environments were caused by the resources available within the host (Cheng et al., 2015), due to a decreased nitrogen quota within cells grown in low nitrogen (Cheng et al., 2015; Maat and Brussaard, 2016). Altered internal quota has also been hypothesized to cause a reduced burst size in cells grown at altered C:P ratios (Clasen and Elser, 2007). In *Thalassiosira weissflogii*, *Dunaliella tertiolecta* (Berges and Falkowski, 1998), and *Chlorella variabilis* NC64A (Cheng et al., 2015), as cells became more limited of nitrogen, their internal C:N ratio increased, hypothetically due to the reduction of nitrogen stores or non-essential proteins (Berges and Falkowski, 1998). This reduced chlorophyll *a* content within the cells and decreased *F*_v_/*F*_m_ as discussed above may prevent viruses from acquiring all of the energy or nitrogen required to produce the large number of viruses per cell. Algae also have increased starch and lipid production (Cheng et al., 2015), and protease activity (Berges and Falkowski, 1998; Frischkorn et al., 2014) under nitrogen stress which may reduce the ability of a successful infection from occurring. More work is needed to understand how stresses influence nutrient and metabolite pools of virocells.

### The Effects of External Nitrogen on the AaV Infection Cycle

The dependence on the external nutrient pools is variable depending on the system and the nutrient. Transitioning *Escherichia coli* grown in a nutrient rich broth to minimal media without various components greatly reduced the T2 phage produced, and the effects of specific nutrient(s) added back partially restored viral production showed the external supplies are required for maximum production of phage (Fowler and Cohen, 1948). Also, adding back various organic phosphorous to phosphorus-starved *Micromonas pusilla* did not decrease the length of time to produce new viruses but did significantly increase the number of viruses produced (Maat et al., 2016b). In contrast, for three marine bacterium-phage systems the majority of the phage genomes were constructed from recycled materials of host origin (Wikner et al., 1993). Moreover, the majority of the nitrogen within the cyanophage S-SM1 was derived from its host, *Synechococcus* WH8102, although amount varied depending on light availability (Waldbauer et al., 2019). To test this in our system, cultures acclimated to a high external ${\text{NO}}_{3}^{-}$ concentration were shifted to environments with decreasing concentrations of external ${\text{NO}}_{3}^{-}$ concentrations post infection. There was no delay in virus production, or a decrease in burst size when cells were moved from a high ${\text{NO}}_{3}^{-}$ external concentration (1.47 mM) to media where there was little (0.0147 mM) or no ${\text{NO}}_{3}^{-}$ added back compared to cultures moved back into the same ${\text{NO}}_{3}^{-}$ external concentration (1.47 mM). We acknowledge using this method, may cause some carry-over of nutrients, but we do believe this method allowed for us to see differences caused by external ${\text{NO}}_{3}^{-}$ concentrations. This can be seen in the control cultures as decreasing external ${\text{NO}}_{3}^{-}$ concentrations caused a significantly reduced end point cell abundance, and a decrease in FL3-H which acts as a proxy for chlorophyll *a* per cell. Cultures with no added ${\text{NO}}_{3}^{-}$ only achieved a final cell abundance 55% that of the cultures with 1.47 mM ${\text{NO}}_{3}^{-}$ added back. This is worth noting as acclimating cultures to even 0.147 mM ${\text{NO}}_{3}^{-}$ (10% that of normal ASP_12_A), had no effect on growth or the infection cycle.

One caveat of this work is that the *A. anophagefferens* cultures are non-axenic. Although there are reports of axenic cultures and methods for their production (Berg et al., 2002), we have been unable to remove the heterotrophic bacteria when cultured on a defined growth medium, suggesting there is the production of a compound required by *A. anophagefferens* not present in ASP_12_A. The use of a defined medium despite the requirement of bacteria, did allow us to know the concentration of ${\text{NO}}_{3}^{-}$ added, and any variability that would exist in a medium with a natural seawater base would not be present. Previous estimates of the number of heterotrophic bacteria in our cultures during late logarithmic growth of *A. anophagefferens* are between 1.8 and 8.1 × 10^5^ colony forming units mL^–1^. The amount of nitrogen found within the heterotrophic community in the cultures therefore could reduce the amount of ${\text{NO}}_{3}^{-}$ *A. anophagefferens* has access to. Using the estimates of nitrogen found within cells in natural and cultural studies (1.6–100 fg cell^–1^) (Fagerbakke et al., 1996), allows for the estimate of amount of nitrogen found within these cells in our cultures (2.88 × 10^5^ to 8.1 × 10^8^ fg of nitrogen). This would account for 1.4 × 10^–4^ to 0.4% of the nitrogen found within the 0.0147 mM ${\text{NO}}_{3}^{-}$ acclimated cultures. Although this is a small amount of the total external nitrogen available, heterotrophic bacteria can be influencing the growth of *A. anophagefferens* in other ways. More work is needed to understand the interactions between *A. anophagefferens* and heterotrophic bacteria, as well as if these bacteria influence the infection cycle in any way.

### Hypothetical Calculations Using Previously Published Data to Assess Nitrogen Requirements for the Infection Cycle to Occur

From the experiments performed in this study, the data support the hypothesis that there is enough nitrogen present within an *A. anophagefferens* cell for the infection cycle to proceed and does not utilize external nitrogen pools. To further support this hypothesis, we performed theoretical calculations from available data about *A. anophagefferens* nutrient pools, C:N:P amounts within the algal virus, PBCV-1, and burst size ranges to assess whether enough nitrogen exists in an infected cell (Table 2).

**TABLE 2:** Estimated use of atoms within an *A. anophagefferens* cell to produce new viruses.

	1 Virus	466 Viruses	2415 Viruses
**Mass in Daltons^A^**			
C	7.4 × 10^8^ Da	3.45 × 10^11^ Da	1.79 × 10^12^ Da
N	2.2 × 10^8^ Da	1.03 × 10^11^ Da	5.31 × 10^11^ Da
P	4.4 × 10^7^ Da	2.05 × 10^10^ Da	1.06 × 10^11^ Da
**Mass in grams**			
C	1.23 × 10^–15^ g	5.73 × 10^–13^ g	2.97 × 10^–12^ g
N	3.65 × 10^–16^ g	1.70 × 10^–13^ g	8.81 × 10^–13^ g
P	7.31 × 10^–17^ g	3.41 × 10^–14^ g	1.77 × 10^–13^ g
**Percentage of atom mass in *A. anophagefferens*^B^**			
C	0.055%	25.63%	132.83%
N	0.11%	51.26%	265.65%
P	0.35%	163.10%	845.25%

*^A^Estimates of mass are based on those of PBCV-1 which a mass of 1 × 10^9^ Da, and a C:N:P ratio of 17:5:1 (Clasen and Elser, 2007). ^B^Based on estimated or calculated masses per A. anophagefferens cell (Gobler et al., 1997).*

The amount of carbon and nitrogen in an *A. anophagefferens* cell has been calculated to be 2.2 × 10^3^ fg cell^–1^ and 3.4 × 10^2^ fg cell^–1^, respectively. The phosphorus concentration has been estimated to be 2.1 × 10^1^ fg cell^–1^ based on estimates using the Redfield ratio (Gobler et al., 1997). The amount of carbon, nitrogen, and phosphorus within an AaV particle are unknown, but these values have been calculated for PBCV-1. We believe this was a good model for AaV as they have a diameter of 1900Å (AaV: data not shown, PBCV-1; Fang et al., 2019), have similar genome sizes [AaV: ∼371 kbp (Moniruzzaman et al., 2014) vs. PBCV-1: ∼331 kbp (Dunigan et al., 2012)], both contain internal membranes (Rowe et al., 2008; Milrot et al., 2017), and have similar capsid structures (AaV: data not shown; PBCV-1; Fang et al., 2019). The C:N:P ratio for PBCV-1 was calculated to be 17:5:1. The ratio was calculated by totaling the C, N, and P found in each macromolecule in the particle, as the percentage of lipids, proteins, and nucleic acids had been previously determined (Clasen and Elser, 2007). As the weight of a single particle is known to be ∼1 × 10^9^ Da (Van Etten et al., 1983), the amount of C, N, and P per particle was calculated.

The burst size of AaV has been shown to be dependent on multiplicity of infection where larger multiplicities of infection produce less viruses per cell (Brown and Bidle, 2014). This is not unique, as it has been shown in other systems (i.e., Van Etten et al., 1982). In this study, two multiplicities of infection were used (∼20 and ∼100 viruses cell^–1^). Using the burst sizes determined with the 1.47 mM ${\text{NO}}_{3}^{-}$ acclimated cultures in both experiments, our results add further evidence that multiplicity of infection influences burst size, as the low multiplicity of infection produced a burst size 5.2 times greater than that of the high multiplicity of infection. The calculated burst sizes in this study are greater than those determined previously by flow cytometry, as Brown and Bidle determined the burst size of AaV to be 191 viruses per cell (2.4–12.6 times less than this study) (Brown and Bidle, 2014). Although the same strain of *A. anophagefferens* and AaV were used, the growth medium used were different. The previous study utilized f/2 media which has a natural sea water base, and different nutrients and metals added to it than ASP_12_A. It is therefore possible this could account for some of the differences. Burst sizes determined in this study are similar to those determined by qPCR in laboratory studies using ASP_12_A (Gann et al., 2020). Differences caused by culturing in different growth media on the infection cycle should be determined. For our calculations we used the average burst size calculated from a high multiplicity of infection of 1.47 mM ${\text{NO}}_{3}^{-}$ cultures (466 viruses per cell), and the average burst size calculated from the low multiplicity of infection of 1.47 mM ${\text{NO}}_{3}^{-}$ cultures (2,415 viruses per cell). These estimates are less than the theoretical maximum number of viruses that could be produced in an *A. anophagefferens* cell. *A. anophagefferens* is between 2 and 3 μm (Gobler and Sunda, 2012), so its volume assuming 2.5 μm is 8.19 × 10^9^ nm^3^, and the volume of an icosahedral with a diameter of 190 nm is 2.17 × 10^6^ nm^3^. Therefore, the theoretical maximum number of particles that can be produced is 3,767 particles per cell. Using these values, around one quarter of carbon and one half of nitrogen within an *Aureococcus* cell would be required to produce a burst size of 466 particles, while over 150% of the phosphorus within the cells would be required. At a high burst size, the quotas of C, N, and P within the cell would not be enough to produce the number of particles (Table 2). These numbers do not consider any virally encoded proteins that are required for production of new progeny but are not packaged. The PBCV-1 genome for instance, encodes for 416 proteins, yet only 148 of them are packaged (Dunigan et al., 2012). Also, not considered in these calculations are if cellular machinery required for the infection needs to continue to be synthesized.

Although, these numbers are crude estimates, it does suggest that the virocell has a strong requirement for external phosphorus, that is not required for nitrogen, to produce a burst size that is similar to those determined in this study. Using the previously published transcriptome of the infected *A. anophagefferens* culture, transporters in the host were evaluated (Moniruzzaman et al., 2018). There were no ${\text{NO}}_{3}^{-}$ transporters over expressed at any point sampled compared to the uninfected controls, and most sugar transporters were either under expressed or did not change (Supplementary Figure 5). In contrast, several inorganic phosphate transporters were over expressed late in the infection cycle, as previously has been reported (Moniruzzaman et al., 2018). These transporters have previously been shown to be upregulated in phosphorus stressed cultures (Wurch et al., 2011).

Differences in uptake of external nitrogen pools in eukaryotic algae seem to depend on the evolution the host virus pair evolved from, and not necessarily on their laboratory growth rate as previously hypothesized (Fowler and Cohen, 1948; Waldbauer et al., 2019). Both *Ostreococcus tauri* and *A. anophagefferens* have similar growth rates when grown on nitrate (Figure 1C; Worden et al., 2004), but the virocell’s reliance on the external environment for nitrogen appears to be different. The lack of change in infection cycle dynamics due to external nitrogen concentration (Figure 3), and the virus-encoded nucleotide recycling genes (Brown and Bidle, 2014; Moniruzzaman et al., 2014), suggest AaV evolved to utilize the internal stores within *A. anophagefferens*. This is further supported by the lack of virally encoded nitrogen or phosphorus transporters. In contrast viruses that infect *O. tauri* encode their own nitrogen transporters which aids the overexpressed host transporters to uptake more external nitrogen than uninfected cells, suggesting this system evolved to utilize external sources (Monier et al., 2017).

### Conclusion

Many harmful algal blooms are terminated by lysis of viruses globally (Brussaard et al., 2005; Vardi et al., 2012), playing important roles in nutrient cycling. The interplay between nutrient availability for the hosts and maximized virus production is not well characterized, and seems to depend on the specific virus-host system (Maat and Brussaard, 2016). Here we have contributed to the growing data about the influence of nitrogen on the infection cycle for an ecologically important virus-host system. Acclimation of cultures to ${\text{NO}}_{3}^{-}$ concentrations that negatively influence growth caused a decreased efficiency of the AaV infection cycle. Our observation suggests that nitrogen-pollution in the early stages of the bloom may influence natural virus-mediated constraints on the bloom but once infection has begun, then nitrogen-loading would have no influence on this control (but may enhance bloom growth). Determining how the infection cycle of AaV responds to the many other nitrogenous species (Berg et al., 2008) may provide further insight into how the host’s metabolism shapes the outcome of interactions with this viral giant.

## Data Availability Statement

The bioinformatic datasets used in this study can be found in NCBI bioproject PRJNA432024.

## Author Contributions

EG and SW designed the experiments. EG and BH completed the experiments. EG, TR, and SW analyzed the data and all authors contributed to the writing and editing of the manuscript.

## Conflict of Interest

The authors declare that the research was conducted in the absence of any commercial or financial relationships that could be construed as a potential conflict of interest.
