# Supplementary material for: Internal Nitrogen Pools Shape the Infection of Aureococcus anophagefferens CCMP 1984 by a Giant Virus
Source: Front Microbiol. 2020 Mar 25;11:492. doi: 10.3389/fmicb.2020.00492 (PMC7109300; doi:10.3389/fmicb.2020.00492)
Supplement: Supplementary file 1 [file Data_Sheet_1.pdf]

Supplemental information to be published online in conjunction with the following:

INTERNAL NITROGEN POOLS SHAPE THE INFECTION OF *AUREOCOCCUS ANOPHAGEFFERENS* CCMP 1984 BY A GIANT VIRUS

Eric R. Gann[1], Brennan J Hughes[1], Todd B. Reynolds[1], and Steven W. Wilhelm[1]

[1]Microbiology Department, University of Tennessee, Knoxville

Telephone: 1-865-974-0665

Fax: 1-865-974-4007

Email: wilhelm@utk.edu

Key words: Mimiviridae, nutrients, brown tide

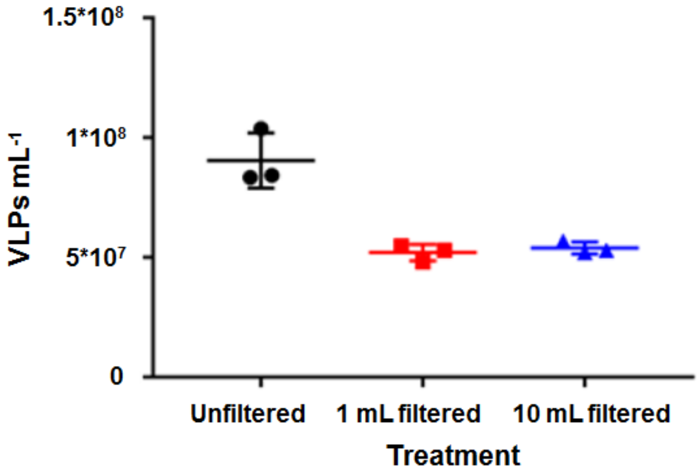

Supplemental Figure 1. Influence of filtering AaV through a 0.45 µm filter. The different treatments were: unfiltered; 1 mL of culture passed through the filter, or 10 mL of culture passed through the filter. Results are from technical triplicates.

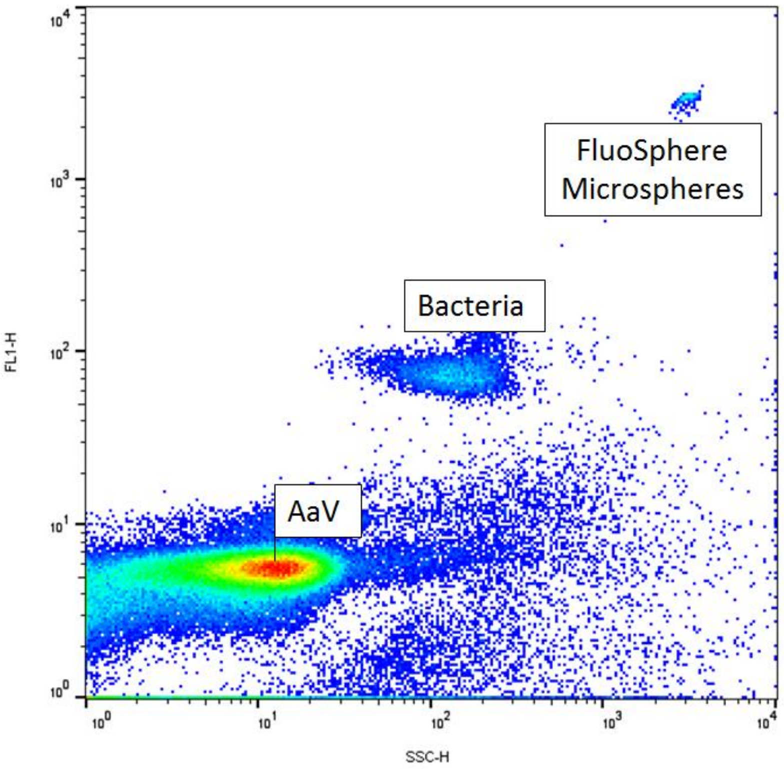

Supplemental Figure 2. Representative FACS Scatterplot of 0.45 µm filtered lysate stained with SYBR green.

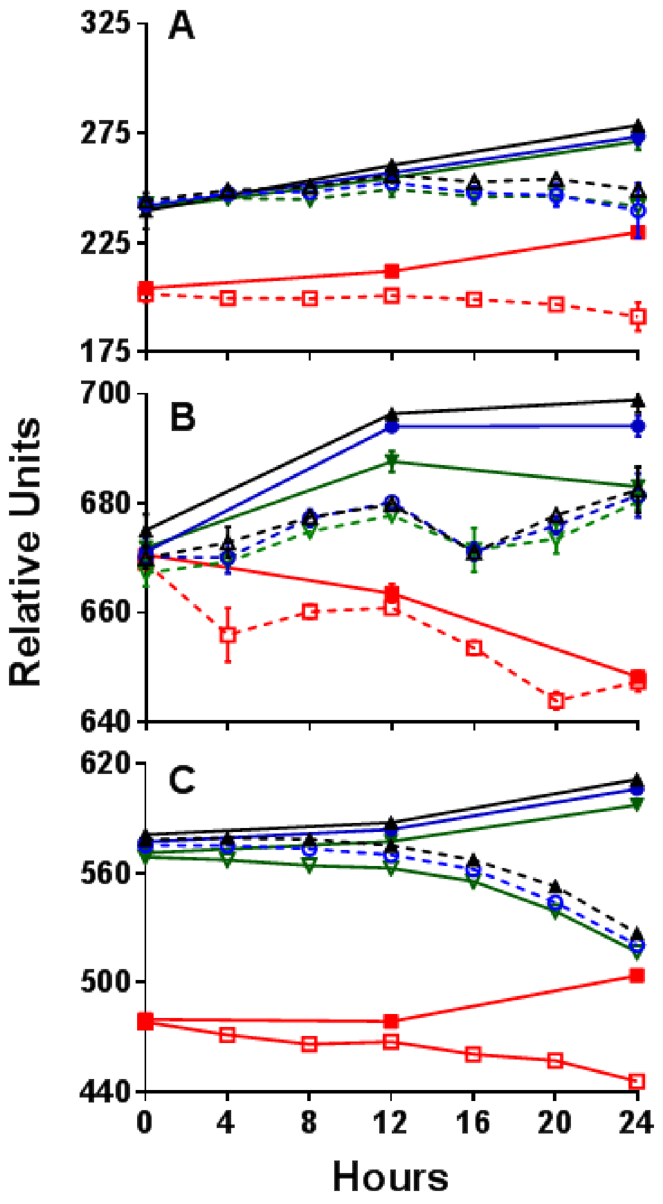

Supplemental Figure 3. Flow cytometry parameters detected over the course of the first 24 hours of the infection cycle. The average of A) FSC-H, B) SSC-H, and C) FL3-H of each sample's gated *Aureococcus* population over time. The different nitrate conditions are represented by the following colors and symbols: 1.47 mM - black upward triangles, 0.735 mM - blue circles, 0.147 mM - green downward triangles, and 0.0147 mM – red squares. Filled in symbols with solid connecting lines represent uninfected controls while open symbols with dashed lines represent infected cultures. Points are for n=5 biological replicates ± SD.

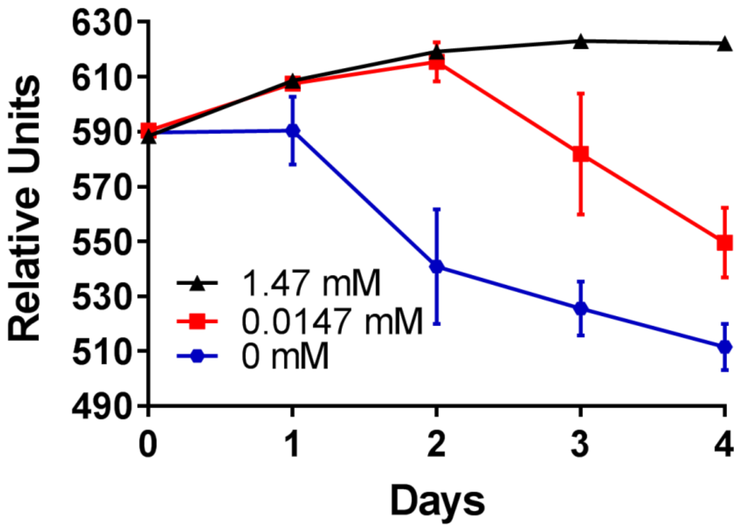

Supplemental Figure 4. FL3-H values of uninfected *Aureococcus anophagefferens* cultures acclimated to 1.47 mM NO_3^-, with different concentrations of NO_3^- added back. The average of FL3-H of each sample's gated *Aureococcus* population over time. The different nitrate conditions are represented by the following colors and symbols: 1.47 mM - black upward triangles, 0.0147 mM – red squares, and 0 mM – blue circles. Points are for n=5 biological replicates ± SD.

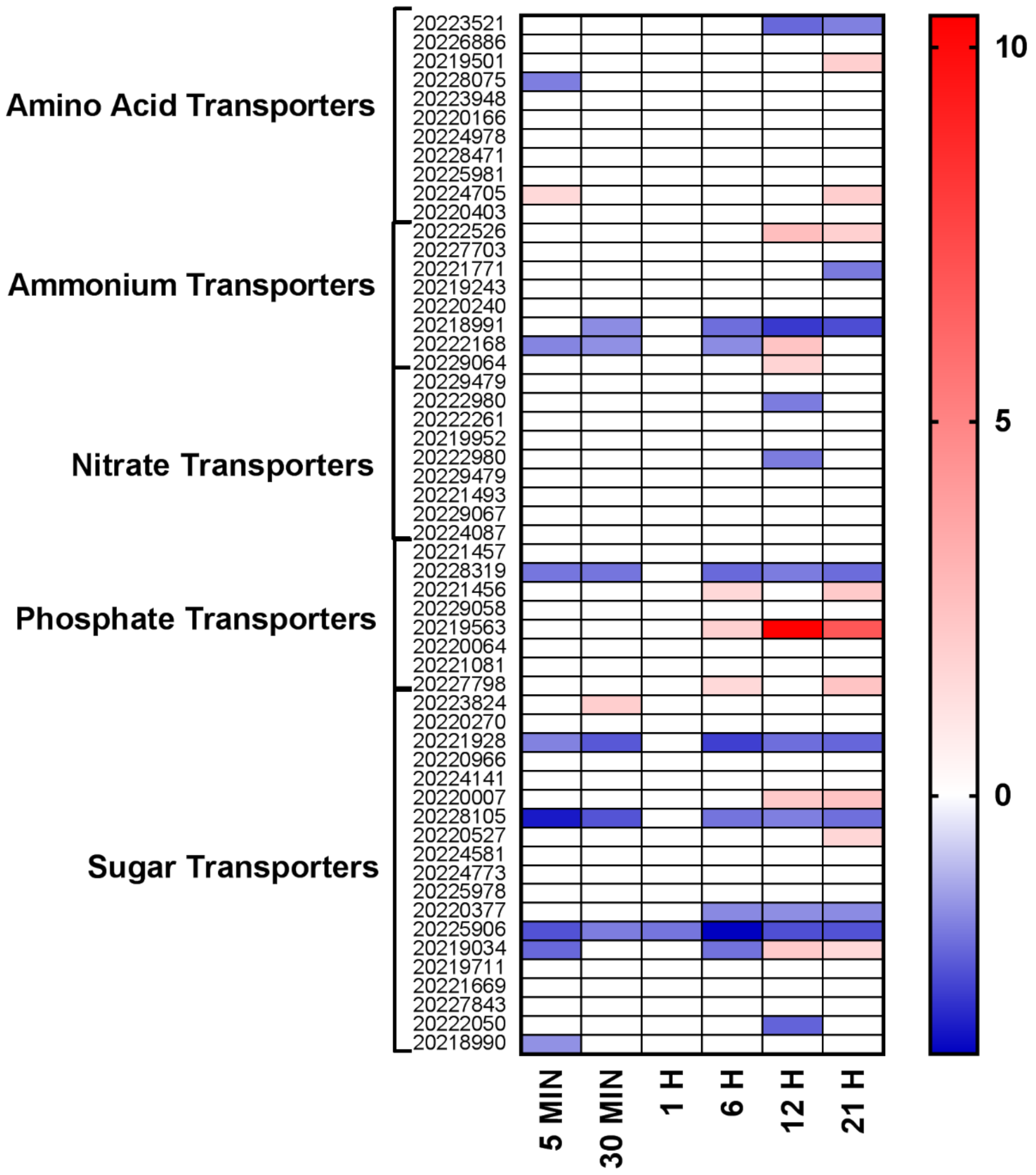

Supplemental Figure 5. Fold change values of transporters found within the *Aureococcus* genome over the course of the infection cycle that are > 1.5 or < -1.5 fold change for infected v. uninfected control cultures with a FDR p-value < 0.05 (Moniruzzaman *et al.*, 2018).

Supplemental Table 1. Adjusted p-values comparing differences in end point cell abundance in an uninfected growth curve based on nitrate concentration (Figure 1A). Adjusted p-values were determined by one-way ANOVA with post-hoc multiple comparisons being adjusted with Tukey's HSD. Red text indicates p-values < 0.05.

End Point Abundances Adjusted p-values (One-way ANOVA; F = 47.31)				
**$[NO_3^{-1}]$**	**1.47 mM**	**0.735 mM**	**0.147 mM**	**0.0147 mM**
**1.47 mM**	-	0.145	0.901	<0.001
**0.735 mM**	0.145	-	0.054	<0.001
**0.147 mM**	0.901	0.054	-	<0.001
**0.0147 mM**	<0.001	<0.001	<0.001	-

Supplemental Table 2. Adjusted p-values comparing differences in doubling time based on nitrate concentration (Figure 1C). Adjusted p-values were determined by one-way ANOVA with post-hoc multiple comparisons being adjusted with Tukey's HSD. Red text indicates p-values < 0.05.

Doubling Times Adjusted p-values (One-way ANOVA; F = 12.1)				
**[NO_3^{-1}]**	**1.47 mM**	**0.735 mM**	**0.147 mM**	**0.0147 mM**
**1.47 mM**	-	0.941	0.053	0.036
**0.735 mM**	0.941	-	0.021	0.085
**0.147 mM**	0.053	0.021	-	<0.001
**0.0147 mM**	0.036	0.085	<0.001	-

Supplemental Table 3. Adjusted p-values comparing differences in different flow cytometry parameters based on nitrate concentration (Figure 1B). Adjusted p-values were determined by one-way ANOVA with post-hoc multiple comparisons being adjusted with Tukey's HSD. Red text indicates p-values < 0.05.

**FSC-H Adjusted p-values (One-way ANOVA; F = 92.98)**				
**$[NO_3^{-1}]$**	**1.47 mM**	**0.735 mM**	**0.147 mM**	**0.0147 mM**
**1.47 mM**	-	0.018	0.012	<0.001
**0.735 mM**	0.018	-	0.992	<0.001
**0.147 mM**	0.012	0.992	-	<0.001
**0.0147 mM**	<0.001	<0.001	<0.001	-
**SSC-H Adjusted p-values (One-way ANOVA; F = 40.16)**				
**$[NO_3^{-1}]$**	**1.47 mM**	**0.735 mM**	**0.147 mM**	**0.0147 mM**
**1.47 mM**	-	0.034	0.034	<0.001
**0.735 mM**	0.034	-	0.869	<0.001
**0.147 mM**	0.034	0.869	-	<0.001
**0.0147 mM**	<0.001	<0.001	<0.001	-
**FL3-H Adjusted p-values (One-way ANOVA; F = 133.9)**				
**$[NO_3^{-1}]$**	**1.47 mM**	**0.735 mM**	**0.147 mM**	**0.0147 mM**
**1.47 mM**	-	0.145	0.079	<0.001
**0.735 mM**	0.145	-	0.812	<0.001
**0.147 mM**	0.079	0.812	-	<0.001
**0.0147 mM**	<0.001	<0.001	<0.001	-

Supplemental Table 4. Adjusted p-values comparing differences in burst size based on nitrate concentrations as determined by one-way ANOVA with post-hoc multiple comparisons being adjusted with Tukey's HSD. Red text indicates p-values < 0.05.

**Burst Size adjusted p-values (One-way ANOVA; F = 18.89)**				
**[NO_3^{-1}]**	**1.47 mM**	**0.735 mM**	**0.147 mM**	**0.0147 mM**
**1.47 mM**	-	0.827	0.678	<0.001
**0.735 mM**	0.827	-	0.240	<0.001
**0.147 mM**	0.678	0.240	-	<0.001
**0.0147 mM**	<0.001	<0.001	<0.001	-

Supplemental Table 5. Adjusted p-values comparing differences in adsorption rate based on nitrate concentrations as determined by one-way ANOVA with post-hoc multiple comparisons being adjusted with Tukey's HSD.

Adsorption Rates adjusted p-values (One-way ANOVA; F = 0.3886)				
**[NO_3^{-1}]**	**1.47 mM**	**0.735 mM**	**0.147 mM**	**0.0147 mM**
**1.47 mM**	-	0.813	0.900	0.776
**0.735 mM**	0.813	-	0.997	>0.999
**0.147 mM**	0.900	0.997	-	0.990
**0.0147 mM**	0.776	>0.999	0.990	-

Supplemental Table 6. Adjusted p-values comparing differences in end point abundance based on nitrate concentrations added back as determined by one-way ANOVA with post-hoc multiple comparisons being adjusted with Tukey's HSD. Red text indicates p-values < 0.05.

End Point adjusted p-values (One-way ANOVA; F = (40.45)			
**[NO_3^{-1}]**	**1.47 mM**	**0.0147 mM**	**0.0 mM**
**1.47 mM**	-	0.004	<0.001
**0.0147 mM**	0.004	-	<0.001
**0.0 mM**	<0.001	<0.001	-

Supplemental Table 7. Adjusted p-values comparing differences in burst size based on nitrate concentrations added back as determined by one-way ANOVA with post-hoc multiple comparisons being adjusted with Tukey's HSD.

**Burst Size adjusted p-values (One-way ANOVA; F = 0.6372)**			
**[NO_3^{-1}]**	**1.47 mM**	**0.0147 mM**	**0.0 mM**
**1.47 mM**	-	0.566	0.988
**0.0147 mM**	0.566	-	0.653
**0.0 mM**	0.988	0.653	-
